# Building a precision medicine infrastructure at a national level: The Swedish experience

**DOI:** 10.1017/pcm.2023.3

**Published:** 2023-02-27

**Authors:** Anders Edsjö, Anna Lindstrand, David Gisselsson, Paula Mölling, Mikaela Friedman, Lucia Cavelier, Maria Johansson, Hans Ehrencrona, Therese Fagerqvist, Tobias Strid, Lovisa Lovmar, Bo Jacobsson, Åsa Johansson, Lars Engstrand, Craig E. Wheelock, Per Sikora, Valtteri Wirta, Thoas Fioretos, Richard Rosenquist, Genomic Medicine Sweden (GMS)

**Affiliations:** 1 Department of Clinical Genetics, Pathology and Molecular Diagnostics, Office for Medical Services, Region Skåne, Lund, Sweden; 2 Division of Pathology, Department of Clinical Sciences, Lund University, Lund, Sweden; 3 Department of Molecular Medicine and Surgery, Karolinska Institutet, Stockholm, Sweden; 4 Clinical Genetics, Karolinska University Hospital, Solna, Sweden; 5 Genomic Medicine Center Karolinska, Karolinska University Hospital, Stockholm, Sweden; 6 Division of Clinical Genetics, Department of Laboratory Medicine, Lund University, Lund, Sweden; 7 Department of Laboratory Medicine, Faculty of Medicine and Health, Örebro University, Örebro, Sweden; 8 Lund University Collaboration Office, Lund University, Lund, Sweden; 9 Innovation Partnership Office, Uppsala University, Uppsala, Sweden; 10 Department of Clinical Pathology, Biological and Clinical Sciences, Linköping University, Linköping, Sweden; 11 Clinical Genomics Linköping, Linköping University, Linköping, Sweden; 12 Department of Clinical Genetics and Genomics, Sahlgrenska University Hospital, Gothenburg, Sweden; 13 Department of Obstetrics and Gynecology, Sahlgrenska Academy, Gothenburg University, Gothenburg, Sweden; 14 Department of Obstetrics and Gynecology, Sahlgrenska University Hospital, Gothenburg, Sweden; 15 Department of Immunology, Genetics and Pathology, Science for Life Laboratory, Uppsala University, Uppsala, Sweden; 16 Department of Microbiology, Tumor and Cell Biology, Centre for Translational Microbiome Research, Karolinska Institutet, Solna, Sweden; 17 Unit of Integrative Metabolomics, Institute of Environmental Medicine, Karolinska Institutet, Stockholm, Sweden; 18 Department of Respiratory Medicine and Allergy, Karolinska University Hospital, Stockholm, Sweden; 19 Clinical Genomics Gothenburg, Science for Life Laboratory, University of Gothenburg, Gothenburg, Sweden; 20 Bioinformatics Data Center, Core Facilities, Sahlgrenska Academy, University of Gothenburg, Gothenburg, Sweden; 21 Department of Microbiology, Tumor and Cell Biology, Clinical Genomics Stockholm, Science Life Laboratory, Karolinska Institutet, Solna, Sweden; 22 School of Engineering Sciences in Chemistry, Biotechnology and Health, Clinical Genomics Stockholm, Science for Life Laboratory, KTH Royal Institute of Technology, Stockholm, Sweden; 23 Clinical Genomics Lund, Science for Life Laboratory, Lund University, Lund, Sweden

**Keywords:** genomic medicine, precision medicine, implementation, national infrastructure

## Abstract

Precision medicine has the potential to transform healthcare by moving from one-size-fits-all to personalised treatment and care. This transition has been greatly facilitated through new high-throughput sequencing technologies that can provide the unique molecular profile of each individual patient, along with the rapid development of targeted therapies directed to the Achilles heels of each disease. To implement precision medicine approaches in healthcare, many countries have adopted national strategies and initiated genomic/precision medicine initiatives to provide equal access to all citizens. In other countries, such as Sweden, this has proven more difficult due to regionally organised healthcare. Using a bottom-up approach, key stakeholders from academia, healthcare, industry and patient organisations joined forces and formed Genomic Medicine Sweden (GMS), a national infrastructure for the implementation of precision medicine across the country. To achieve this, Genomic Medicine Centres have been established to provide regionally distributed genomic services, and a national informatics infrastructure has been built to allow secure data handling and sharing. GMS has a broad scope focusing on rare diseases, cancer, pharmacogenomics, infectious diseases and complex diseases, while also providing expertise in informatics, ethical and legal issues, health economy, industry collaboration and education. In this review, we summarise our experience in building a national infrastructure for precision medicine. We also provide key examples how precision medicine already has been successfully implemented within our focus areas. Finally, we bring up challenges and opportunities associated with precision medicine implementation, the importance of international collaboration, as well as the future perspective in the field of precision medicine.

## Impact statement

Implementation of precision medicine in healthcare has been made possible thanks to the rapid development in high-throughput sequencing technologies coupled with new types of targeted drugs being introduced, hence realising personalised treatment and care. Genomic Medicine Sweden (GMS) was formed to enable an equal and resource-efficient implementation of precision medicine across the country. Through regional centres for genomic medicine at all Swedish university hospitals and a common infrastructure for data sharing, GMS has built up the capacity for precision diagnostics/medicine in rare diseases, cancer, infectious diseases and complex diseases. In this article, we summarise our experience with implementing precision medicine at a national level, discuss remaining challenges and opportunities, as well as future directions in this rapidly advancing field.

## Introduction

The introduction of high-throughput sequencing (HTS) technologies at a broader scale more than 10 years ago provided entirely new research tools for the discovery of molecular events associated with disease (Koboldt et al., 2013). This led to a rapid increase of novel disease genes associated with rare inherited diseases (Gilissen et al., 2012; Stranneheim and Wedell, 2016), but also unravelled the genomic landscape in major cancer types (Ley et al., 2008; Stratton et al., 2009; Bailey et al., 2018; PCAWG-Consortium, 2020). In parallel, new types of therapies were developed that target disease-driving molecular alterations or key cellular pathways and/or processes with higher precision than before (Malone et al., 2020; Tsimberidou et al., 2020; Wasterlid et al., 2022). These developments led to the fast introduction of genomic technologies, such as whole-exome (WES)/genome (WGS) and gene panel sequencing, into clinical routine diagnostics for rare diseases, cancer and infectious diseases (Zehir et al., 2017; Lindstrand et al., 2019; Kobras et al., 2021; van der Sanden et al., 2022; Walter et al., 2022). In this way, genomic-based techniques paved the way for advanced molecular profiling as a basis for individualised treatment and follow-up, that is, precision medicine (Stranneheim and Wedell, 2016; Malone et al., 2020; Rosenquist et al., 2022; Wasterlid et al., 2022; Yang et al., 2022).

One of the first national genomic initiatives, Genomics England, was launched in 2013, with an ambition to sequence 100,000 genomes in patients with rare diseases or cancer (Turnbull, 2018; Investigators et al., 2021; Degasperi et al., 2022). After successfully finishing the project in 2018, genomic technologies were implemented in routine healthcare within National Health Services (NHS) through seven Genomic Medicine Services centres across the country (Turnbull et al., 2018), while also embarking on new pioneering projects, such as the new-born genome program and the population screening program for early cancer detection using liquid biopsy. Today, national precision medicine strategies/initiatives have been launched in many countries worldwide (more than 30 initiatives in Europe only), for example, the Danish National Genome Center, Genomic Medicine France 2025, Australian Genomics and Singapore’s National Precision Medicine program (Lethimonnier and Levy, 2018; Turnbull et al., 2018; Saunders et al., 2019; Stark et al., 2019; Kong et al., 2022; Stenzinger et al., 2022). In other countries, such as Sweden and Germany, with regionally organised healthcare, academia and healthcare have instead joined forces to establish regionally distributed genomic/precision medicine centres with a close national coordination (Fioretos et al., 2022; Stenzinger et al., 2022).

In this review, we will describe our efforts in Sweden to build a national infrastructure for the implementation of precision medicine, while also highlighting key disease areas for which precision medicine approaches are being implemented. Finally, we will discuss important challenges that we need to address to enable equal access to precision medicine nationally and internationally as well as future directions in this rapidly evolving field.

## Building a national infrastructure for precision medicine

**Figure 1.**
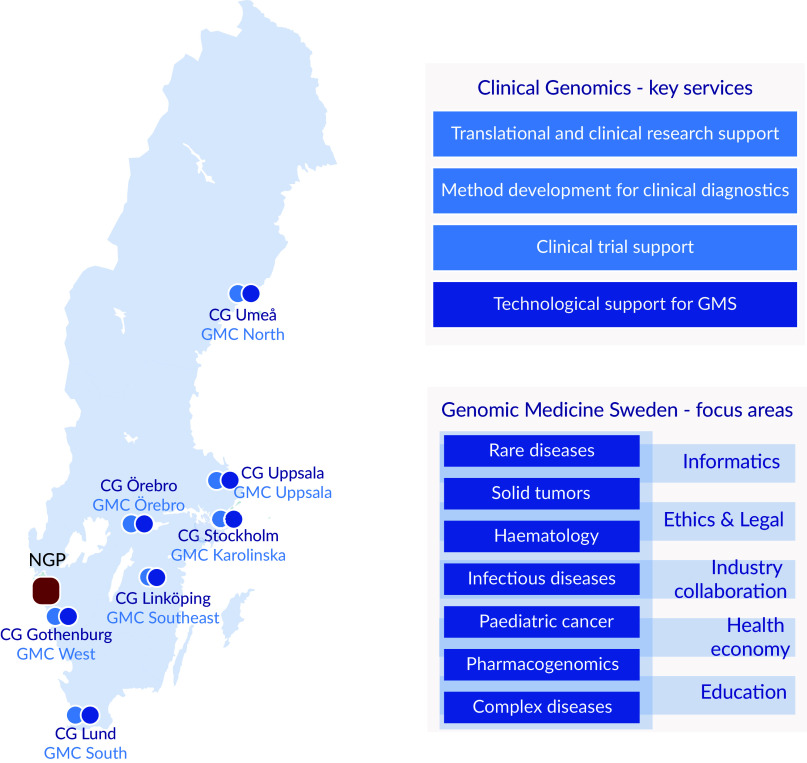
Regional distribution, key services and focus areas. Clinical Genomics (CG) units are located at the seven universities with medical faculties, and Genomic Medicine Centres (GMCs) at the seven university hospitals in Sweden. The National Genomics Platform (NGP), located in Western Sweden (Region Västra Götaland), is a highly competent data lake linked to a dynamic scale out high performance computing cluster. CG provides expertise and services to the research and industrial community, and to GMS. GMS currently encompasses seven diagnosis-specific working groups and five working groups supporting the GMS infrastructure.

One important development in Sweden was the establishment in 2010 of an infrastructure for molecular biosciences, *Science for Life Laboratory* (SciLifeLab) that today provides a wide range of high-throughput technologies through ten technology platforms at a national level. One of these platforms, the *Clinical Genomics* platform, is focusing on the development, adaptation and implementation of genomic technologies for translational research and clinical utility. To secure national coverage, Clinical Genomics nodes are located at all seven universities with medical faculties in Sweden (Figure 1; Fioretos et al., 2022). In 2017, the Clinical Genomics platform initiated the formation of a national infrastructure for genomic medicine/precision medicine which was named *Genomic Medicine Sweden* (GMS). A *Swelife*-funded pre-study phase allowed for key stakeholders to lay the foundation of the overall organisation of GMS and the establishment of national working groups coordinating activities in key areas relevant for precision medicine. Funded by an implementation grant from the Swedish Innovation Agency *Vinnova*, GMS was officially inaugurated in 2018.

In Sweden, the responsibility for healthcare is divided between the national government, the 21 healthcare regions and the municipalities, where the regions have the responsibility to organise healthcare in such a way that all citizens have access to good and equal healthcare. To overcome any possible regional differences and to be directly linked to healthcare, GMS is therefore organised as a regionally distributed infrastructure. The implementation of precision medicine is performed through the seven Genomic Medicine Centres (GMCs) that have been established at each of the university hospitals with close links to the technology-driven SciLifeLab Clinical Genomics node at each site, which provides a critical technological backbone for GMS (Fioretos et al., 2022).

The core partners of GMS are the seven university healthcare regions and the seven universities with a medical faculty. In addition, GMS works closely together with SciLifeLab, patient organisations, industry and government agencies. GMS is led by a national steering board, which consists of seven members appointed by each of Sweden’s seven university healthcare regions (usually the research director), two members jointly appointed by the remaining 14 regions, seven members appointed by each of seven universities with medical faculties (usually the dean of the faculty), two members from industry, two members from patient organisations as well as adjunct members from SciLifeLab and Biobank Sweden (Stenzinger et al., 2022). The strong national anchoring of the steering board, especially in regional healthcare, has been a key success factor in the implementation of genomic medicine in Swedish healthcare. The GMS operation is led by a director, together with the GMS collaboration office and the GMS management group with representations from all core partners.

The overarching aims of GMS are to (i) implement genome sequencing in healthcare for improved diagnostics and provide equal access to personalised treatment, (ii) establish a national IT-infrastructure and associated knowledge-bases to advance precision medicine in Sweden, (iii) increase the use of genomics and health data for research and innovation, (iv) increase participation in clinical trials and (v) implement genomics in prevention, diagnostics and patient stratification of complex diseases (in more detail in the *GMS Strategy Plan 2021–2030*). These goals are closely linked to the ambitions of the Swedish government’s *life science strategy.*

GMS is organised in seven disease-specific national working groups: rare diseases, solid tumours, haematological malignancies, childhood cancer, infectious diseases/microbiology, complex diseases, as well as pharmacogenomics (Figure 1). The disease-specific working groups are in turn supported by working groups in data management and informatics, health economy, ethical and legal issues, industry collaboration and education.

## The National Genomics Platform

Because of the regionalised IT infrastructures at the university hospitals in Sweden, where information is kept in multiple non-interconnected systems, GMS informatics working group is building a dedicated infrastructure for data handling and storage, that is, the National Genomics Platform (NGP), also to standardise and organise genomic data across the GMCs (Fioretos et al., 2022; Stenzinger et al., 2022). The central part of the NGP is a data lake for genomic data and associated clinical metadata. By creating a unified infrastructure, it becomes feasible to enforce standards for data structuring as well as creating national analysis pipelines. Organising data within a joint national infrastructure also simplifies data availability for research purposes, as well as data interoperability through a single standardised interface.

**Figure 2.**
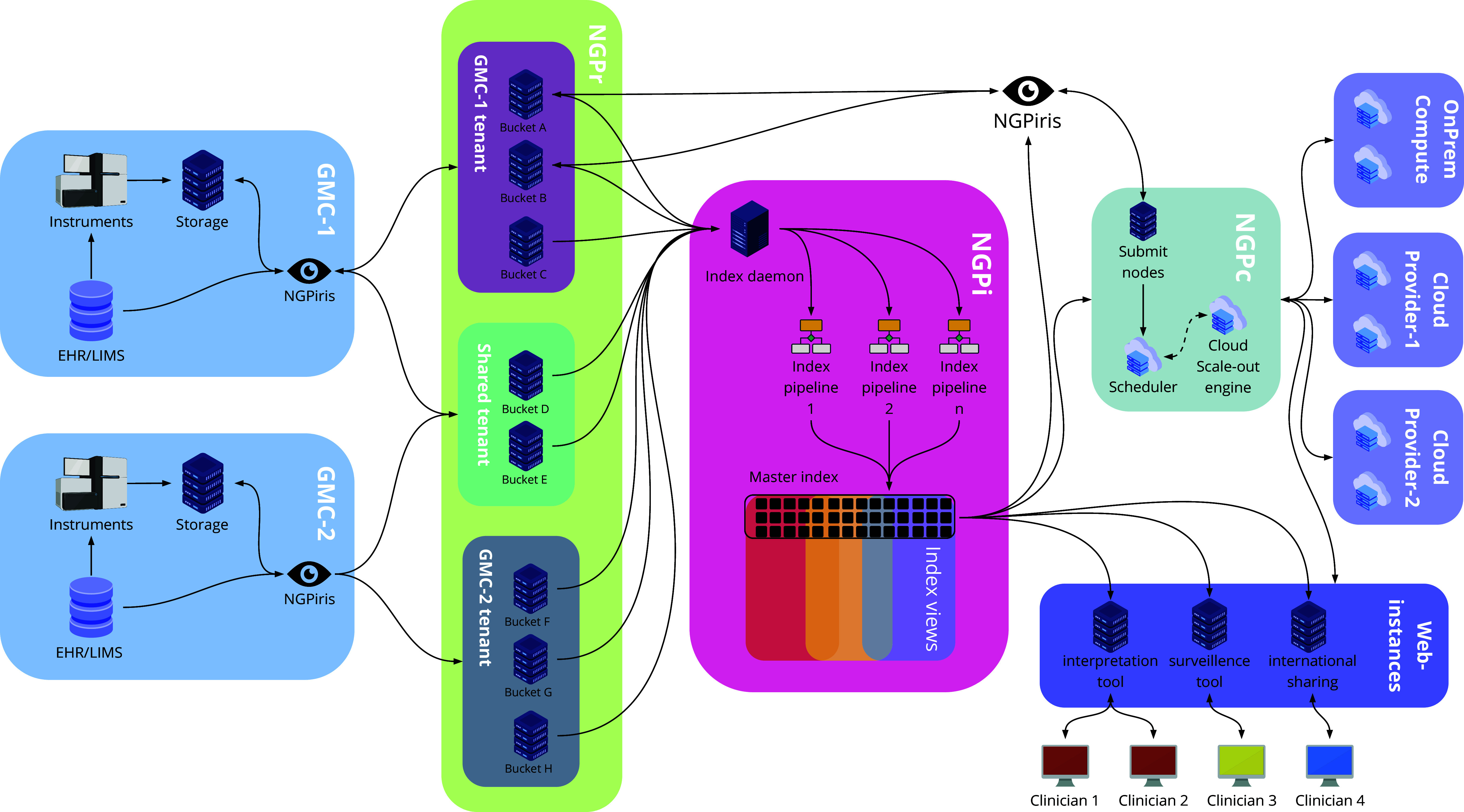
Schematic view of the National Genomics Platform. The platform is divided into three distinct parts covering storage (NGPr), indexing and metadata analysis (NGPi) and data processing (NGPc). Each GMC has its own tenant within the platform creating the possibility of logical separation between centres. The separation persists in the indexing layer, allowing a fine-grained control over what metadata is shared between centres. The indexing layer can then serve as a back-end for both interpretation tools and national and international data sharing. Data processing can be either local on-prem or provisioned on-demand in one or multiple cloud providers’ platforms.

The platform is divided into three subparts for storage, indexing and processing (Figure 2).

The central storage within the NGP consists of an advanced object storage that is hosted in three separate geographic locations within Region Västra Götaland (located southwest in Sweden). All seven GMC are linked to the central storage through the Swedish hospital network. Using the GMS-developed middleware NGPiris, data and metadata can be culled from local LIMS and storage systems and uploaded to the central platform using the S3 protocol, ensuring data security and standardisation. Within the platform, data is logically separated by a tenant system where each GMC/university hospital has full control over their own data, including access and user control. Access rights can be set on individual objects, allowing targeted data sharing between GMCs.

For data to be utilised for interpretation and sharing, the NGP platform contains a powerful indexing engine based around Apache SOLR. Data is indexed in near real-time using multiple indexing pipelines, extracting information from various types of data files. Data points from multiple files are combined into metadata objects held within the index. Since the metadata object structure of the NGP is non-static, new information can be retrieved and indexed from the object store as new needs are identified. Access to the index can be tailored using index views that limit access to certain metadata points and certain indexes for certain applications, creating fine-grained access control. In other words, searches can be limited by what the respective user/application is allowed to “see” within the index and sensitive information can be hidden.

The NGP uses a combination of existing on-prem compute resources as well as cloud-based infrastructure to provide resource scaling. This is achieved through a cloud scale-out engine, based on the open-source Tortuga-project, and by engaging Swedish-owned cloud providers for scale-out, ensuring high security and compliance with Swedish law.

## Implementation of precision diagnostics/medicine in healthcare

In the following sections, we will describe our efforts in implementing precision medicine within our focus disease areas, rare diseases, cancer, infectious diseases and complex diseases.

### Rare diseases

Rare diseases constitute a large number of different disorders, of which the vast majority are severe genetic chromosomal or single-gene disorders. Although individually rare, these disorders are collectively common with an estimated population prevalence of 3.5–6% (Nguengang Wakap et al., 2020). The most important factor for effective treatment and preventive measures is a molecular diagnosis. With an exact diagnosis, patients can be offered personalised management, healthy relatives may receive carrier testing and prenatal diagnosis is possible.

**Figure 3.**
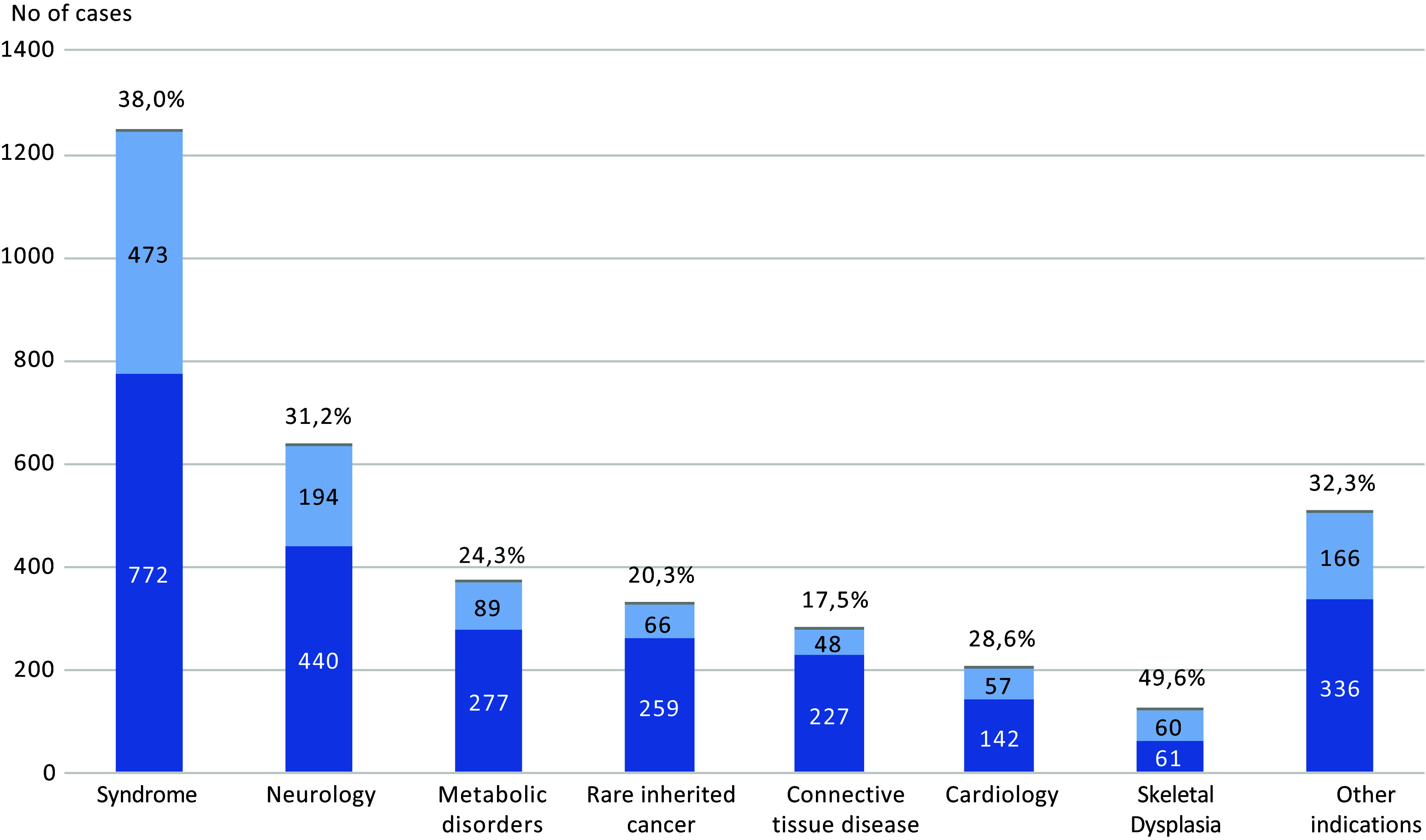
Number of analysed patients using WGS analysis in specific disease groups of rare disease. Positive (light colour) and negative (dark colour) genetic findings with corresponding diagnostic yield above each bar. Based on WGS analysis performed at three GMCs during 2021.

To identify the disease-causing pathogenic variants in rare genetic diseases, WGS is emerging as a powerful first-line screening test regularly used in routine diagnostics (Lionel et al., 2018; Investigators et al., 2021; Stranneheim et al., 2021). The diagnostic yields vary between different disease categories and centres, depending on inclusion criteria and whether a trio or singleton approach was used (Figure 3). For individuals with intellectual disability, WGS yields diagnostic rates between 30 and 40% (Investigators et al., 2021; Stranneheim et al., 2021; Lindstrand et al., 2022; van der Sanden et al., 2022), which is comparable or even superior to the combined yield after multiple genetic tests such as WES, chromosomal microarray and targeted gene panels (Lindstrand et al., 2022; van der Sanden et al., 2022), while shortening the time to diagnosis substantially. Furthermore, WGS has the potential to improve the clinical yield even more in the future by adding analysis of novel genetic causes of disease, both coding and non-coding.

To maximise the diagnostic yield multiple variant types need to be called and interpreted clinically. As such, there is a need to optimise the analysis pipelines, databases and interpretation tools. Furthermore, the diagnosis of patients is still dependent on linking the genetic variants to clinical symptoms. This is reflected in the need for multidisciplinary collaborative environments, where laboratory specialists interact with both bioinformaticians and clinical specialists, a necessary prerequisite to implement genomics in healthcare (Investigators et al., 2021; Stranneheim et al., 2021).

For rare disease patients, the diagnostic work is executed at the seven GMCs. As of 1 January 2022, more than 10,000 rare diseases patients have been analysed by WGS in Sweden. Of those, 22% were analysed as trios and 78% as singletons and the overall diagnostic yield was 36% and 33%, respectively. The projection is that over 5,000 additional individuals will undergo testing in 2022 resulting in a rapidly growing number of patients receiving a diagnosis.

One of the prioritised areas in the GMS rare diseases working group is to improve diagnostics by building a clinical database for genotype-phenotype matching within the NGP, as well as to establish classified variant databases and aggregated variant databases for improved variant prioritisation. This would also provide an important resource for Mendelian disease discovery as well as understanding other types of inheritance such as digenic, and mutational burden. To include as many individuals as possible we are also working on an electronic informed consent process at the national level.

The strategy for WGS-based diagnostics of rare disease patients has initially focused on well-known variant categories such as single-nucleotide variants and insertions/deletions, followed more recently by detection of structural variants, including copy-number variants (CNVs), repeat expansions and mtDNA (Dolzhenko et al., 2019; Lindstrand et al., 2019; Stranneheim et al., 2021; Ibanez et al., 2022). To develop the WGS-based precision diagnostics further, two national projects are conducted aiming at validating and implementing RNA-sequencing for confirmation of WGS findings and to improve understanding on regulatory, non-coding genomic variation and, secondly, long-read sequencing for improved resolution and calling of structural variants in challenging regions such as low-complexity regions.

### Solid tumours

Solid tumours make up around 90% of the 62,000 cancer cases diagnosed yearly in Sweden (Socialstyrelsen, 2020). Of these, roughly 10,000 cases are currently analysed with focused gene panels, mainly for treatment-predictive purposes. Non-small cell lung cancer is the leading indication with up front reflex testing at all seven GMCs and almost 20 targeted drugs available. Patients with colorectal carcinomas and malignant melanomas are also commonly tested, along with a number of other solid tumours tested in select cases.

**Figure 4.**
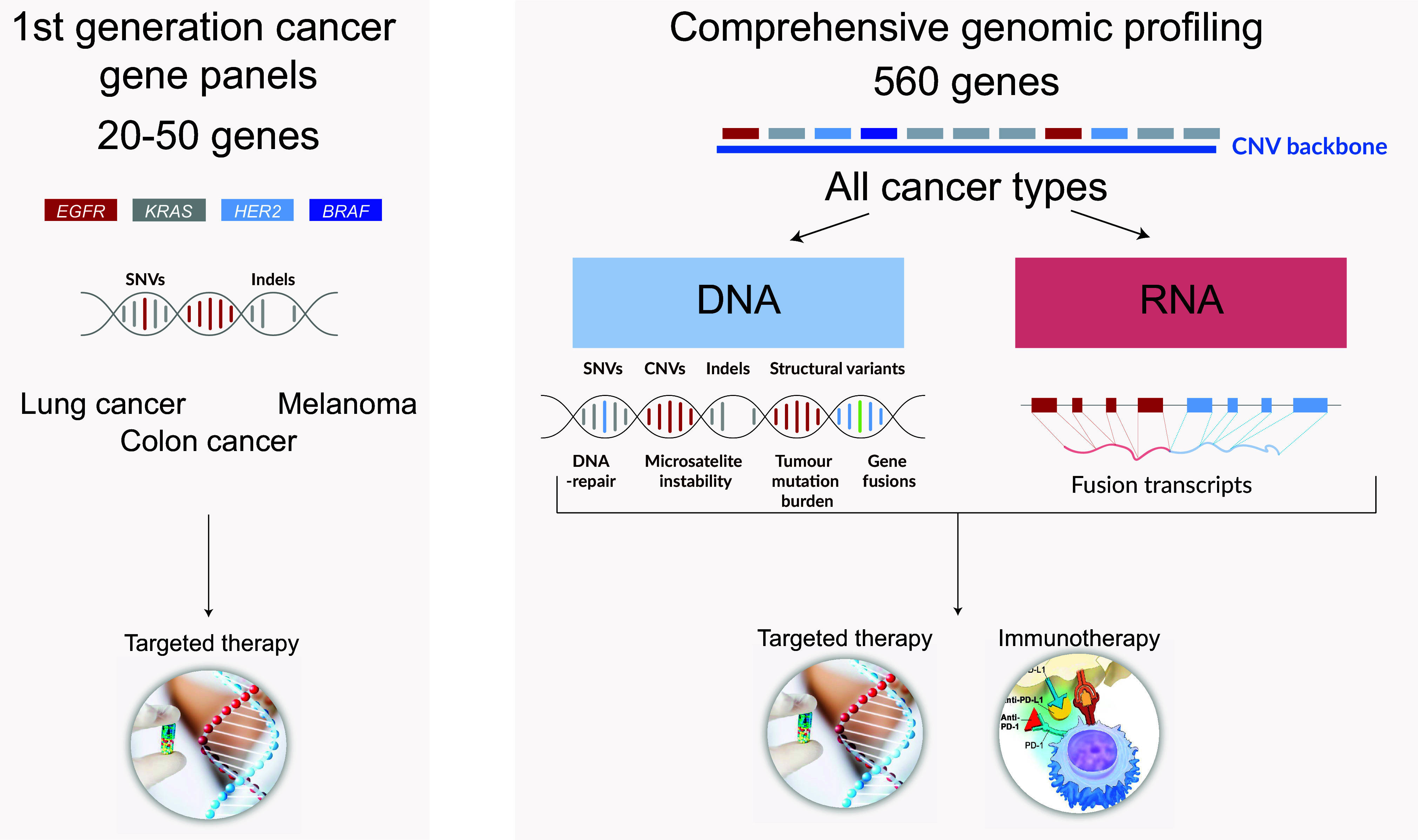
Genomic profiling of solid tumours illustrating first- and second-generation gene panels. CNV, copy-number variant; indels, insertions and deletions; SNVs, single nucleotide variants.

Given the rapidly increasing number of biomarkers and the need to assess structural variations and to generate assessments of more complex biomarkers, such as microsatellite instability (MSI), homologous recombination deficiency (HRD) and tumour mutational burden (TMB) based on mutational signatures (Malone et al., 2020; Mosele et al., 2020), a national gene panel (GMS560) for comprehensive genomic profiling of routine clinical samples was given the highest priority in the GMS solid tumour working group (Figure 4). The need was also underscored by the tumour-agnostic indications for MSI and NTRK fusions and ongoing clinical trials on additional tumour-agnostic drugs targeting mainly gene fusions, indicating an increased need for comprehensive genomic profiling (Le et al., 2015; Hong et al., 2020).

During the design phase, a structured review of current indications and biomarkers relevant for clinical studies was supplemented with input from the national clinical consensus groups for cancer treatment. In addition, variants relevant for tumour biology identified in the TCGA consortium were added for scientific purposes and for future-proofing of the design (Sanchez-Vega et al., 2018). Finally, a number of pharmacogenomic targets were included to enable identification of genetic aberrations impacting drug susceptibility. The responsibility for the development and validation of the various modules in the panel has been divided between the seven participating sites, using the combined resources of all involved clinical laboratories, GMCs and SciLifeLab Clinical Genomics nodes. To ensure equity of care and harmonised results for complex biomarkers, a modular bioinformatics solution has been developed as part of the national effort.

The implementation of the panel has gained considerable support via pilot projects for precision medicine funded by the Swedish Ministry of Health and Social Affairs. Starting in 2021, the pilots for solid tumours included the addition of a module for HRD testing, gene panel testing for breast cancer patients with stage 4 disease, as well as a pilot for tissue and liquid biopsy testing in lung cancer as the latest addition. The pilots have served as a valuable means to involve clinicians in each field and made it possible to discuss priorities as well as important issues on how best to convey results.

Liquid biopsies, the use of blood samples to test for biomarkers normally analysed in tissue samples, have the potential to address several important clinical questions (Markou et al., 2022). Currently used to detect resistance mutations, monitoring of treatment response, molecular characterisation in cases with a shortage of available material and a means to visualise molecular heterogeneity, are all potential applications in the clinical setting. A future next step includes early detection of cancer with the potential to improve treatment outcomes (Crosby et al., 2022).

With widened indications for targeted treatments in additional malignancies and increased need to follow treatment response and resistance development, the testing can be expected to double within the coming years. Another important trend is the focus on the diagnostic importance of molecular characterisation, mirroring the increased weight given to molecular alterations in the WHO classifications of malignant disease (WHO-Classification-of-Tumours-Editorial-Board, 2021a, 2021b).

Another challenge has been to establish precision medicine trials for cancer to ensure that the data generated from the comprehensive molecular profiling can be translated into treatment and that novel treatments can be evaluated. In this work, GMS and its founding partners has joined forces with Zero Vision Cancer, MEGALiT, SciLifeLab and several relevant authorities in the field of life science in Sweden to form the “Test Bed Sweden for Clinical Trials and Implementation of Precision Health in Cancer Care.” Through this network, Nordic and European collaborations have been established and support for joint projects have been secured, allowing for work on setting up drug repurposing studies in additional European countries and refining molecular diagnostics and molecular tumour boards.

### Haematological malignancies

Haematologic malignancies represent highly molecularly heterogeneous disorders with an increasing number of disease-driving alterations being described and incorporated into classification and treatment guidelines (Alaggio et al., 2022; Arber et al., 2022; de Leval et al., 2022; Dohner et al., 2022; Khoury et al., 2022). Traditionally, these disorders are divided into myeloid and lymphoid neoplasms depending on the cellular lineage affected by the disease-driving alterations.

**Figure 5.**
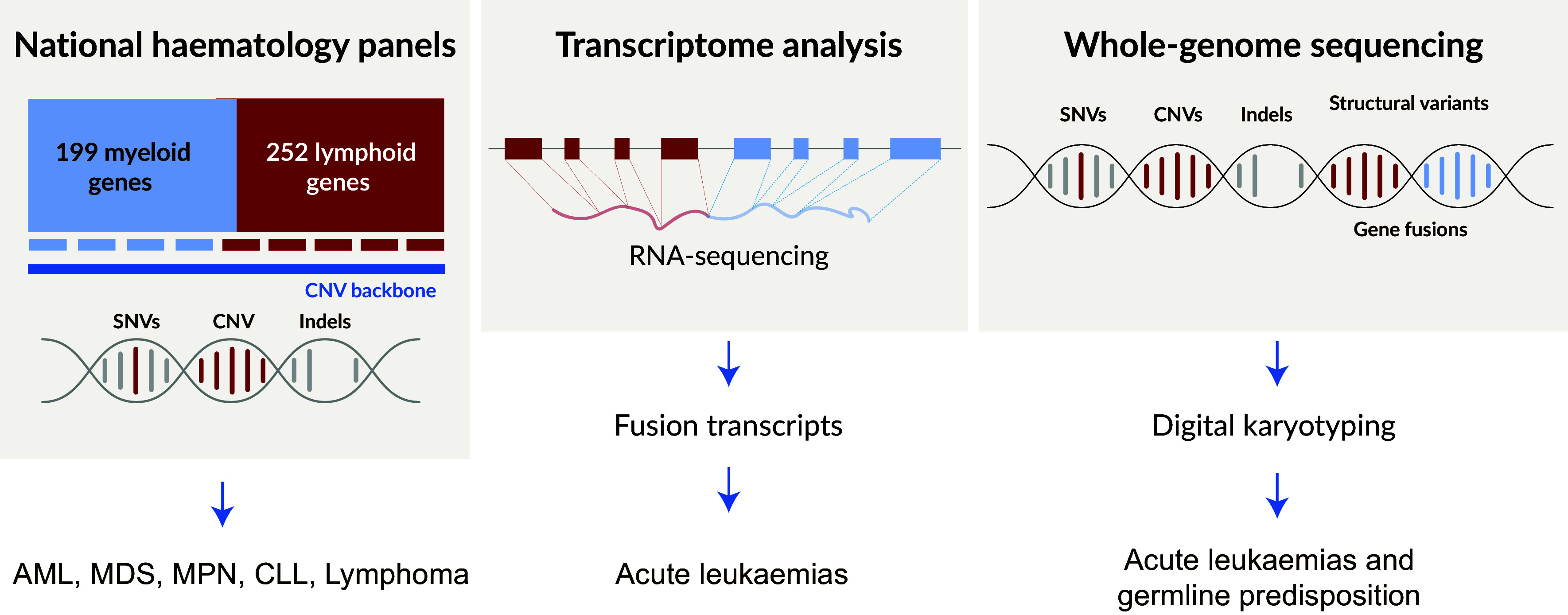
National strategy for precision diagnostics in haematological malignancies. ALL, acute lymphoblastic leukaemia; AML, acute myeloid leukaemia; CLL, chronic lymphocytic leukaemia; CML, chronic myeloid leukaemia; indels, insertions and deletions; MDS, myelodysplastic syndrome; MPN, myeloproliferative neoplasias; SNVs, single nucleotide variants.

Early on, GMS recognised that in an evolving landscape with new genomic markers being described at a rapid pace in haematological malignancies, the design of a comprehensive and highly flexible gene panel was needed. Apart from allowing the detection of a high number of single nucleotide variants (SNVs), smaller insertions/deletions and CNVs, there was a need to seamlessly incorporate new diagnostic markers in the gene panel design. GMS decided to use a targeted capture-based approach, allowing an even and deep coverage of disease variants, as well as the detection of smaller insertions/deletions (Figure 5). The first gene panel introduced in clinical diagnostics was the GMS-Myeloid Gene Panel (GMS-MGP), which allows the detection of variants in 199 genes together with a backbone of single nucleotide polymorphisms (SNPs), allowing the detection of CNVs at a 10-Mb resolution, as chromosomal imbalances form an important basis for risk-stratification and treatment prediction in haematological malignancies. The gene panel also includes genes that are associated with hereditary predisposition to haematological malignancies, as well as pharmacogenetic genes of importance for drug susceptibility. The analytical and clinical performance of the GMS-MGP was recently investigated in a national interlaboratory study, showing a high concordance of the results between the participating GMCs (Orsmark-Pietras et al., manuscript in preparation). To date, >5,000 tests have been performed in a clinical diagnostic setting using the GMS-MGP.

Using a similar capture-based protocol as the GMS-MGP, a lymphoid gene panel (GMS-LGP) was designed allowing the detection of variants in 252 genes. The design of the second version is ongoing for improved detection of CNVs relevant for lymphoid malignancies, and will also include structural variation and immunoglobulin/T-cell receptor genes.

In a national study, GMS is currently also evaluating if WGS in combination with whole-transcriptome sequencing (WTS) can replace current standard of care (SoC), including multiple, often time-consuming and costly diagnostic genetic tests (Figure 5; Berglund et al., 2022). In this study, 700 cases of adult and paediatric acute myeloid leukaemia (AML) and acute lymphoblastic leukaemia (ALL) are subjected to WGS and WTS and the results are compared to SoC using chromosome banding, fluorescence *in situ* hybridization (FISH), SNP-arrays, directed molecular tests and the GMS myeloid and lymphoid gene panels. In parallel to evaluating the ability of WGS and WTS to replace SoC as a diagnostic tool, health-economy studies are performed within the study. Other upcoming studies within GMS will include the evaluation of single-cell technologies for clinical applications, a rapidly advancing field (Pfisterer et al., 2021), as well as methods allowing ultrasensitive detection of measurable residual disease of critical importance for monitoring treatment effects and to predict disease relapse at an earlier stage (Chen et al., 2022; de Leval et al., 2022).

### Childhood cancer

In children, leukaemia is the most common malignancy, while CNS tumours dominate among solid neoplasms. However, there is also a myriad of rare childhood cancers. Many of these rare entities have diagnostic markers at the genomic level, such as somatic gene fusions, point mutations or characteristic patterns of copy-number imbalances. Also, within diagnostic entities, genetic profiling is often used for prognostication and risk stratification. The diversity of diagnostic entities combined with the many types of clinically relevant genomic aberrations have made it difficult to set up comprehensive gene panel-based approaches for childhood cancer. The situation is further complicated by the constant discovery of new genomic markers that have to be included in diagnostic platforms. Taken together, this situation prompted a broad technical approach when considering a standardised clinical genomics pipeline for children with cancer.

The GMS Childhood Cancer working group resolved this issue by nationwide implementation of WGS coupled with WTS as part of the routine clinical work-up of paediatric malignancies. To achieve unbiased geographic coverage, patients from all of Sweden’s six paediatric oncology centres, of which some are very small, were included from the start of implementation in 2021. To create resilience by redundancy and to disseminate expertise though the country, WGS and WTS for childhood cancers was established at the GMCs at the large university hospitals in Stockholm, Gothenburg and Lund, as well as for leukaemia in Uppsala. The nationwide coverage and the fact that all paediatric malignancies are includible, makes our effort distinct from previous studies having focused on high-risk patients or large tertiary care centre cohorts (Wong et al., 2020; Newman et al., 2021; Trotman et al., 2022).

**Figure 6.**
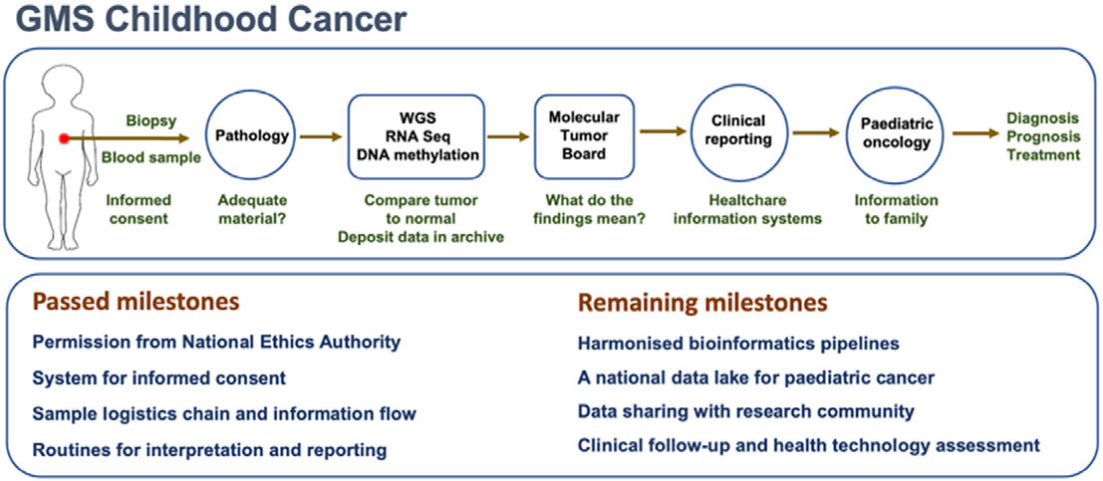
Infographics of the GMS Childhood Cancer pipeline. The upper panel outlines the main steps for each patient’s sample and the resulting information: (1) inclusion based on informed consent and tumour cell content in biopsy >40%, (2) WGS of tumour DNA (minimum 90×), normal sample DNA (30×), and tumour RNA-sequencing, (3) filtering of tumour WGS data against normal sample WGS data to identify somatic mutations, (4) further filtering of non-synonymous coding variants against a flexible gene list of somatic mutations of clinical importance in childhood cancer, also including potential druggable targets, (5) fusion gene capture from RNA-sequencing data, (6) creation of whole genome profiles of copy numbers and allelic states, (7) discussion of findings at a molecular tumour board and (8) formulation of a written report, added as a complement to the standard pathology report. The bottom panel itemises passed milestones and future plans.

Irrespective of where the patient was diagnosed and cared for primarily, the same steps were taken from inclusion to clinical reporting (Figure 6). In a parallel project, germline variants of clinical importance are identified from normal sample WGS data (peripheral blood leukocytes for solid tumours, skin biopsies for leukaemias). Also, for CNS tumours, DNA methylation classification is performed on a regular basis. At the date of writing, around 150 children in Sweden have gone through this clinical pipeline. In the vast majority of cases, WGS and RNA sequencing provided information that corroborated, revised or refined the original diagnosis (manuscript in preparation).

### Microbiology/infectious diseases

The importance of implementing precision medicine in healthcare to prevent and treat infectious diseases is underscored by the drastic emergence of both antimicrobial resistance (AMR) and pandemics that affects and kills millions of people globally every year (Marani et al., 2021; Antimicrobial-Resistance-Collaborators, 2022; Fink et al., 2022). A functional infrastructure for pandemic preparedness, including AMR monitoring, should encompass a broad portfolio of genetic technologies that can be employed into the healthcare system. This requires new technical solutions and IT structures for data processing. The SARS-CoV-2 pandemic has accelerated this, where GMS has been setting up a joint automated workflow for bioinformatics analysis of SARS-CoV-2 on the NGP. The platform will also be the basis for other pathogenic microorganisms in future, where a nationwide implementation of WGS enables fast and reliable identification and typing of microorganisms, that is, bacteria, viruses, parasites and fungi. Methods and results can easily be shared in real time between regions affected by outbreaks, both to map the spread of infectious diseases but also to detect emergent spread of antibiotic resistance. International comparisons can thus be facilitated, which has been shown to be of great importance during the pandemic (Pires et al., 2022).

Putting the genetics into clinical practice to identify SNPs related to the severity of disease or antibiotic resistance will also pave the way for precision medicine in the managing and treatment of infectious diseases. The work within GMS has started with the bacterium multi-resistant methicillin *Staphylococcus aureus* (MRSA) where sequencing data from nine laboratories, generated with different sequencing technologies, have been compared regarding quality and different analysis pipelines to guide further harmonisation with the ultimate aim to set up one joint workflow on the NGP.

Genomic techniques such as metagenomics (sequencing of all DNA or RNA within a sample) is one way to identify and characterise a microorganism directly in a sample. The metagenomic approach offers rapid diagnostics and a possibility to detect unknown pathogens without culturing (Purushothaman et al., 2022). As part of the pandemic preparedness in Sweden, GMS has in collaboration with the SciLifeLab Pandemic Laboratory Preparedness capability, worked on establishing an assay and analysis pipeline for clinical metagenomics that is currently being established nationally and will be available on the NGP.

The same metagenomics technique is also used to characterise the human microbiome that will be of great importance to predict risk and treatment outcomes in various diseases including chronic inflammation and cancer. An integrative approach combining data sources will provide taxonomic profiles, diversity and functional annotations in such samples. The implementation of machine learning is needed to increase our capacity to classify and predict clinical outcomes and will be an important tool in precision medicine and infectious disease management (Peiffer-Smadja et al., 2020).

### Complex diseases

While progress has been made in precision medicine approaches in cancer and rare diseases, most common diseases, including allergies, asthma, cardiovascular disease, diabetes, neurologic diseases, psychiatric disorders and rheumatic diseases, pose more of a challenge. These diseases are referred to as complex because most patients do not carry established single-gene mutations with large effect sizes, and the disease risk is instead explained by a complex set of genomic variations in combination with lifestyle and environmental factors (Wheelock and Rappaport, 2020; Franks et al., 2021). For instance, while a strong genome-wide genetic correlation has been identified between asthma and allergic diseases (Lionel et al., 2018), attempts to explain the incidence of obstructive lung disease or allergy solely by genetics have not been successful. Collectively, complex diseases account for ~70% of global deaths and the majority of healthcare costs (GBD-Disease-Injury-Incidence-Prevalence-Collaborators, 2017). To address this challenge, GMS has formed a working group to identify common complex diseases where a genetic link has been identified, but where this information is not yet used optimally in clinical practice.

During the last few years, the possibility of combining genetic associations across the genome into a polygenic risk score (PRS) has been investigated for many complex diseases (O’Sullivan et al., 2022). For example, in coronary artery disease, it has been shown that a high PRS confers an equal risk as monogenic effects (Khera et al., 2018), and a higher risk than conventional risk factors. For preventive purposes, many international organisations already recommend to estimate the 10-year cardiovascular risk for adult patients 40 to 75, and including the PRS will contribute to increased accuracy of the current risk prediction tools (O’Sullivan et al., 2022). PRS has also been used to further refine risk assessment among women with suspected predisposition to breast cancer (Mavaddat et al., 2019; Lakeman et al., 2020), and a web-based risk assessment tool, CanRisk Web Tool, based on PRS, family history and rare pathogenic variants in cancer susceptibility genes has been developed (Carver et al., 2021).

However, for most PRS the precision is too low to be clinically relevant for individual patients and there are no clear guidelines available on how to translate PRS to the benefit of individual patients. For example, the variance in asthma liability explained by 22 distinct genome-wide-significant variants is estimated to be as low as 3.5% (Demenais et al., 2018), and 12 common genetic variants associated with airflow obstruction and risk of chronic obstructive pulmonary disease only explain about 1.5% of the phenotypic variance (Hobbs et al., 2017). For many complex traits (Yengo et al., 2022), a dramatically larger sample size is therefore needed in the GWAS studies before a PRS will be of high enough precision to identify high- or low-risk individuals of complex diseases. However, it is worth highlighting that environmental risk factors, and environmental exposure, remain the predominant drivers for development of several complex diseases, including, for example, obstructive lung disease.

It is clear that substantially more research is required to be able to take genomic precision medicine into the clinic and many such initiatives are underway. There is a need to include other omics technologies, such as proteomics and metabolomics (Beger et al., 2016; Duarte and Spencer, 2016), which offers the advantage of being closest to the current phenotype of the patient, into precision medicine efforts to predict, diagnose and ultimately treat and prevent complex diseases. Following this approach, there have been recent efforts to investigate the utility of biomarker-based approaches to stratify asthmatics for treatment with biologics and patients with atrial fibrillation to receive oral anticoagulation (Benz et al., 2021; Israel et al., 2021).

In summary, while the healthcare organisation of Sweden is conducive to expanding precision medicine treatment approaches into the area of complex diseases, there remains a general lack of knowledge about how molecular information can be used to benefit the diagnosis, prevention and treatment of patients with complex diseases. It is most probable that eventual precision treatment efforts for complex diseases will involve a combination of genetic and other molecular information to create biomarker panels for optimising patient treatment strategies.

## Challenges and opportunities working at a national level

In a regionally distributed healthcare setting, aligning work habits, analytical methods and processes and bioinformatic pipelines is essential to harmonise data generation nationally and fully enable the utilisation of the potential in the data. As an example, for cancer diagnostics, where molecular profiling of solid tumours, haematological malignancies and childhood cancer has evolved as separate fields, work has been initiated to create molecular assays utilising a common technical approach, as well as establish a modular bioinformatic framework addressing the needs of each disease area.

The NGP is of key importance to enable national data sharing, including databases of genetic variation, and real-time analysis for healthcare professionals, but will also create a trusted research environment for secondary use of genomic data for the research community and other stakeholders in the life science sector. However, to fully use and benefit from the technical solutions for data sharing established in the NGP, the current Swedish legislation relating to the secondary use of health data requires changes. To address this, an *inquiry* was recently initiated by the Swedish government.

Strategic collaboration with industry and other societal stakeholders is crucial to achieve the long-term goals of GMS and thus to enable the development and implementation of precision medicine. The core partners of GMS and the national trade associations of the life science industries have agreed on a framework for collaboration to provide guidance on legal issues and create improved conditions for collaboration for the mutual benefit of the partners, thus contributing to accelerate the transformation of the healthcare system towards precision diagnostics/medicine with the patient at centre stage. Industry collaboration is challenged by the organisational structure of GMS constituting 14 partners within academia and healthcare, partly governed by different laws and regulations. The rapid development of knowledge, and making research and healthcare intertwined, may also be challenging with respect to the format of collaboration. In the short-term perspective, increased collaboration with industry will accelerate development and innovation, based on joint learnings and active knowledge sharing between organisations.

Another important area concerns education and training, as well as increasing awareness. Here, all relevant stakeholders across the precision medicine ecosystem need education about genomic/precision medicine, including healthcare professionals, patients and the society at large. As a consequence of the fast technological development and shift in healthcare, an increased dialogue concerning patient/individual integrity and ethical issues, for example, in sharing healthcare data will be important. GMS is actively collaborating with the medical profession and patient associations to provide different types of educational activities, including webinars, workshops and online courses. Moving forward cooperation with patient representatives, from planning and design to evaluation and follow-up, will increasingly be important parts of GMS’ work. There is also a need to incorporate the precision medicine concept into undergraduate and graduate programs as well as to start new educational programs, for example, in clinical bioinformatics. Here, GMS will be an important partner.

Finally, to build a sustainable infrastructure for precision medicine, a strong national commitment is necessary in order to secure long-term financing. In Sweden, a *roadmap* for precision medicine and advanced therapy medicinal products was recently released by one of the government’s strategic cooperation programs that proposed the establishment of a national infrastructure for precision medicine where the state and healthcare regions join forces in a long-term partnership which in turn will enable equal access to precision medicine for all citizens across the country.

## Future directions

Currently, a main driver of precision medicine is high-throughput sequencing and there is a substantial potential for additional clinical benefit from further developing these techniques. Additional indications and complex biomarkers can be expected to make their way into clinical routine (Alexandrov et al., 2013). New analyses are expected to be added both for blood with fractionation of, for example, thrombocytes, extracellular vesicles and circulating tumour cells as well as cerebrospinal fluid, urine and other body fluids in which nucleic acids can be enriched. The methods used for analysing circulating tumour cells can also be expected to be useful for single-cell analyses, adding new dimensions of molecular heterogeneity (Lin et al., 2021; Pfisterer et al., 2021).

In the coming years, other types of information and technologies can be expected to become increasingly clinically relevant, such as long-read sequencing, optical mapping of structural variations, multimodal single cell and spatial omics and genome-wide methylation analyses. All these new modalities need to be taken all the way from discovery to clinical implementation and routine diagnostics. To facilitate this, the SciLifeLab Clinical Genomics platform and GMS have established a research implementation framework (Fioretos et al., 2022), and other high-end technology platforms within SciLifeLab are likely to further promote Swedish precision in the near future using a similar strategy.

Regardless of the information modality, the vast amounts of data generated necessitates new approaches, including machine learning and artificial intelligence, to be able both to discern patterns and to find the individual details of future clinical relevance. The data from diagnostics and clinical studies also need to be linked to and cross-referenced with health data in quality registries and electronic health records, adding to the complexity and need for new data processing solutions. Here, a recently established large-scale national initiative in *Data-Driven Life Sciences* by SciLifeLab, funded by the Knut and Alice Wallenberg Foundation, is likely to promote the capability in Sweden to harness the massive amounts of data being generated for improved precision medicine research and diagnostics.

Furthermore, the in-depth data and targeted drugs of precision medicine stratify patients in smaller groups than handled by traditional clinical trials, calling for novel approaches to clinical study design. This is an important task, involving clinicians and diagnostic people alike, and the development of the field is driven by international collaborations to pool the data needed.

Finally, as evidenced by ongoing active collaboration between European precision medicine initiatives, the best way forward will be to form cross-border and cross-disciplinary collaborative networks to foster and create the healthcare of tomorrow for the benefit of our patients and the society at large.
