# Supplementary material for: Building a precision medicine infrastructure at a national level: The Swedish experience
Source: Camb Prism Precis Med. 2023 Feb 27;1:e15. doi: 10.1017/pcm.2023.3 (PMC10953755; doi:10.1017/pcm.2023.3)
Supplement: Peer review history

# Reviewer report: Review: Building a precision medicine infrastructure at a national level: The Swedish experience — R0/PR2

**DOI:** 10.1017/pcm.2023.3.pr2

**Authors:** Theodora Katsila

**Affiliations:** Institute of Chemical Biology, National Hellenic Research Foundation, Greece

*Comments to Author*: Edsjo et al present GMS via regional centres for genomic medicine at all Swedish university hospitals and a common infrastructure for data sharing with the aim of precision diagnostics and/or precision medicine in rare diseases and complex traits, such as cancer and infectious diseases. This is a well written manuscript with a clear rationale. Challenges and opportunities are clearly and eloquently described. The authors include their views and comments on current strategies based on their national experience. Such findings are of benefit to the readership in particular when it comes to implementation

That said it would be nice for the authors to elaborate on the tasks that could be taken by information technologies to ease translation to the clinic considering limitations and future opportunities.

# Editor report: Recommendation: Building a precision medicine infrastructure at a national level: The Swedish experience — R0/PR3

**DOI:** 10.1017/pcm.2023.3.pr3

**Affiliations:** Faculty of Medicine and Dentistry, Queen Mary University of London Faculty of Medicine and Dentistry, United Kingdom of Great Britain and Northern Ireland

No accompanying comment.

# Editor report: Decision: Building a precision medicine infrastructure at a national level: The Swedish experience — R0/PR4

**DOI:** 10.1017/pcm.2023.3.pr4

**Affiliations:** Institute of Cardiovascular & Medical Sciences, University of Glasgow College of Medical Veterinary and Life Sciences, United Kingdom of Great Britain and Northern Ireland

No accompanying comment.
